# Spatiotemporal dynamic relationships and simulation of urban spatial form changes and land surface temperature: a case study in Chengdu, China

**DOI:** 10.3389/fpubh.2024.1357624

**Published:** 2024-06-28

**Authors:** Ling Jian, Xiaojiang Xia, Yuanqiao Wang, Xiuying Liu, Yue Zhang, Qianchuan Yang

**Affiliations:** ^1^College of Geography and Planning, Chengdu University of Technology, Chengdu, China; ^2^Key Laboratory of Digital Mapping and Land Information Application, Ministry of Natural Resources, Wuhan, China; ^3^Research Center for Human Geography of Tibetan Plateau and Its Eastern Slope, Chengdu University of Technology, Chengdu, China

**Keywords:** local climate zone, urban landscape pattern, space–time cube, ordinary least squares model, urban planning

## Abstract

Exploring the spatiotemporal dynamic evolution of local climate zones (LCZ) associated with changes in land surface temperature (LST) can help urban planners deeply understand urban climate. Firstly, we monitored the evolution of 3D urban spatial form in Chengdu City, Sichuan Province, China from 2010 to 2020, used the ordinary least squares model to fit the dynamic correlation (DR) between the changes in urban spatial patterns and changes in LST, and revealed the changes of urban spatial patterns closely related to the rise in LST. Secondly, the spatiotemporal patterns of LST were examined by the integration of the Space–Time Cube model and emerging hotspot analysis. Finally, a prediction model based on curve fitting and random forest was integrated to simulate the LST of study area in 2025. Results show the following: the evolution of the urban spatial form consists of three stages: initial incremental expansion, midterm incremental expansion and stock renewal, and late stock renewal and ecological transformation. The influence of the built environment on the rise of LST is greater than that of the natural environment, and the building density has a greater effect than the building height. The overall LST shows a warming trend, and the seven identified LST spatiotemporal patterns are dominated by oscillating and new hotspots patterns, accounting for 51.99 and 11.44% of the study area, respectively. The DR between urban spatial form and LST varies across different time periods and built environment types, whereas the natural environment is always positively correlated with LST. The thermal environment of the city will warm up in the future, and the area affected by the heat island will shift to the central of the city.

## 1 Introduction

The rapid development of urbanization has changed not only the spatial structure of cities but also the urban thermal environment, which is facing many threats and challenges as a result (1, 2). The changes in urban spatial form and land surface temperature (LST) exhibit spatial correlation and heterogeneity, and overlooking this relationship may lead to spatial inequality in climate change mitigation (3), further affecting human health and social welfare. Local Climate Zones (LCZ) are defined as “areas spanning several hundred meters to several kilometers on a horizontal scale, characterized by uniform surface cover, structure, material, and human activity (4),” and serve as an effective standard method for distinguishing the thermal environmental heterogeneity of urban spatial forms (5). Understanding the dynamic evolution of urban spatial forms and the assessment of urban thermal environments based on LCZ is important to propose differentiated planning strategies to cope with them.

Differences in urban spatial form and layout can lead to the spatiotemporal differentiation of LST, and optimizing the urban spatial form can effectively mitigate the urban heat island effect (UHIE) (6). Previous studies have shown that the spatial distribution of land use and land cover (LULC) has an enormous influence on the urban thermal environment (7, 8). However, current LULC studies still lack support for local climate change research due to simplified classification and inability to detect thermal intensity changes within cities at finer spatial scales. Zoning practice is an effective means to bridge the gap between urban climate research and urban planning (5). Therefore, Stewart proposed the scheme of LCZ (4). Currently, studies related to LCZ mainly include on LCZ scientific mapping using different classification methods and combinations of multiple data sources (9, 10) and research on UHIE issues based on LCZ (11, 12). In terms of urban climate research, due to the heavy, challenging workload of LCZ mapping, most of the studies are on the classification of LCZ and UHIE assessment at specific times, and the analysis of the evolution on the spatiotemporal sequences is still in its infancy, although relevant research results have been obtained in some regions, such as Fuzhou (13) and Dalian (14), China, the Yangtze River Delta region (15), and Bangkok, Thailand (16). However, the analysis of LCZ changes in multi-temporal sequences is still limited, and the relationship between changes in urban spatial form and LST changes is analyzed from a static perspective. Although the existing studies on the relationship between urban landscape pattern and LST help us understand the link between the two, the results of these studies are uncertain and contradictory. For example, green spaces with great patch density can alleviate LST in Zhuhai, China (17) and Southeast Asian cities (18), but the results are opposite those in a study in Beijing (19). In Wuhan, water shape complexity is negatively associated with LST and positively associated with mountainous cities like Chongqing (20). Some studies have suggested increasing green space coverage can alleviate LST (21), but some scholars have found that a decrease in green space coverage increases the cooling effect (22). The reasons for these inconsistent results may include: a lack of multi-temporal dynamic changes analysis in urban research data, making it difficult to accurately reflect the relationship between landscape patterns and LST at a specific time; LULC classification lacks a fine scheme, ignoring the effects of changes in the form, height, and density of buildings and vegetation on LST; The scale and size of the study area vary according to pixel size. Therefore, a scientific method still needs to be found to understand the relationship between urban spatial forms and LST.

Line graphs and thematic maps of spatial distribution are commonly used for studying spatiotemporal characteristics. Line graphs present the general trend of time change, and thematic maps demonstrate information on the spatial distribution of research objects. Yu (23) utilized spatial statistical analysis to reveal the spatiotemporal patterns of the regional heat island in the Pearl River Delta Metropolitan Region. Previous methods were based only on temporal (1D) or spatial (2D) information, and considering the visualization of spatiotemporal information simultaneously is difficult (24). Space–Time Cube (STC) and emerging hotspot analysis is a new spatiotemporal data model that integrates geographic data into a 3D cube, in which the x and y axes represent spatial location and the z axis represents time. The STC model can show the spatiotemporal dynamics of the overall evolutionary characteristics of the data, overcoming the discretization and discontinuity in time and space of traditional spatiotemporal analysis. This integrated approach provides a new opportunity for long-term Earth observation and environmental monitoring. The traditional hotspot analysis method can only output hotspots with different confidence levels, whereas the emerging hotspot analysis method can combine time dynamics to output different types of 17 cold and hot spot patterns, which are new, consecutive, intensified, persistence, oscillating, dispersed, diminishing, and historical cold and hot spots, as well as one that is devoid of significant features (25). Currently, some studies use this method to integrate spatiotemporal information for spatiotemporal data reconstruction and apply long-term evolution monitoring analysis to urban informatics, traffic analysis, and other fields, such as COVID-19 epidemic monitoring (26) and traffic flow and accident analysis (24, 27). However, this integration is rarely applied in the field of remote sensing analysis. Building a STC based on multi-temporal LST raster data, while considering the changes in space and time, helps analyze the evolution trends of cold and hot spots in LST, which aids in overcoming the issues of spatiotemporal discretization and discontinuity in traditional remote sensing analysis.

In the study of urban thermal environments, the accurate prediction of LST is essential to understanding the future UHIE and developing appropriate mitigation strategies. In previous research on LST predictions, multiple linear regression (MLR) models have been used to identify the influencing variables of the thermal environment (28, 29). After identifying the relationship between the dependent and independent variables, the MLR model was applied to make predictions. However, MLR models do not adequately capture the nonlinear relationships between variables, whereas the Random Forest (RF) model, based on machine learning methods, is widely used to handle nonlinear interactions and offers high simulation accuracy (30). The prediction results of previous LSTs relied on a single prediction model and lacked the combination of multiple prediction models (31, 32). The results of a single prediction model can only demonstrate the high or low accuracy of the model overall, which may lead to the prediction results of some locations not being the optimal choice. Therefore, a multi-model integration approach can provide an effective solution that combines the prediction results of different models, utilizes the advantages of different models while compensating for the shortcomings between models, and selects the best prediction for each location by accurately evaluating the accuracy, thus improving the overall prediction performance of the model.

In summary, we explores the relationship between urban spatial form changes and LST and predicts the LST in 2025. This study aims to answer the following questions: What are the characteristics of the urban spatial form changes, and how do the changes affect the spatiotemporal differentiation of LST? How much does the change in the urban landscape pattern affect LST, and what is the relationship between them? How is the spatial distribution of urban spatial pattern changes that are closely related to the rise of LST identified? What are the characteristics of combining different prediction models in the application of LST prediction in Chengdu? The results of this paper will help deepen our understanding of the effects of dynamic changes in landscape composition and the configuration of refined urban spatial form on LST and provide a reference for the accurate prediction of future LST studies.

## 2 Study area and data

### 2.1 Study area

**Figure 1:**
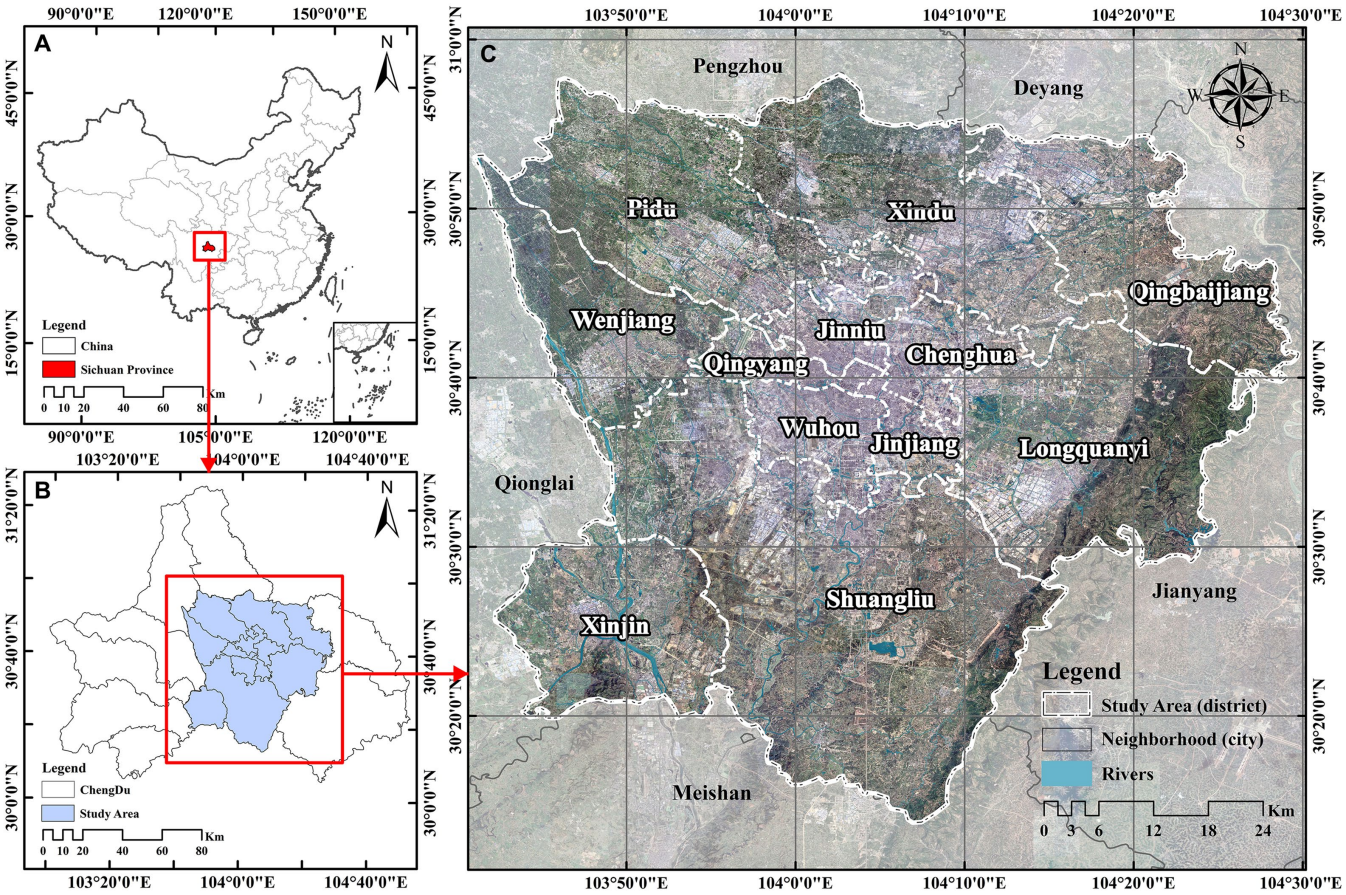
Location map of the study area: **(A)** the location of Chengdu City in China, **(B)** the location of the central districts in Chengdu City, and **(C)** a satellite image of the central districts of Chengdu City.

The study area is the central districts of Chengdu City, Sichuan Province, China (103°40′E–104°30′E. 30°05′N–31°0′N), which includes 12 administrative districts with a total area of 4007.07 km^2^ (Figure 1). This range not only include the main urban construction area of Chengdu, while the surrounding area also covers most of the natural environment elements, and can meet the needs of the LCZ delineation scheme. The study area is the hinterland of the Chengdu alluvial Plain backed by the Longmen and Longquan Mountains, which represents a typical city on flatlands. The Chengdu city is located in the transition zone from the Northwest Sichuan Plateau to the Sichuan Basin, with a long river network and a developed water system. Humid subtropical climate brings sufficient heat and rainfall to the city, but its low altitude and special geographic and climatic conditions lead to frequent impairment to the city’s ventilation conditions. According to the monitoring data from various meteorological stations in Chengdu from 2007 to 2015, the annual average wind speed (0.93 m/s) in the administrative district is low, and the frequency of static winds is as high as 40% (33). Such ventilation conditions are not only detrimental to the diffusion of pollutants, but also exacerbate the UHIE. Chengdu’s rich historical and cultural heritage has created a unique urban spatial and morphological pattern that has become rich and complex after a rapid urbanization. Exploring the relationship between the spatial form changes and LST changes in Chengdu will help provide a basis for future urban land use structure adjustments to cope with heat mitigation.

### 2.2 Data and source

The study mainly used Landsat series satellite remote sensing data combined with Google Earth Pro, Baidu Street View maps, and field investigation data as the sources of data. Landsat satellite have the advantages of long coverage time, free availability, and high spatial resolution, so Landsat remote sensing data with 30 m spatial resolution originated from the United States Geological Survey website^1^ were used to monitor the spatiotemporal evolution of LCZ and LST in Chengdu from 2010 to 2020. All Landsat images were collected during the daytime in the summer and autumn (May to September) of each year (overpass time was 11:33 a.m. Beijing time), and the screening principle was that the cloud cover was less than 10%. The selected Landsat data and related information are shown in Table 1. Because the cloud cover in the Landsat images in the study area in 2012 was high, it may bias the LST inversion. Therefore, the imaging data from 2011 were used as a substitute. Additionally, we used average air temperature observation data from the same dates as the remote sensing images to validate the effectiveness of LST. This data was sourced from the Basic Meteorological Element Dataset of National Ground Meteorological Stations in China.^2^ The study area includes six basic meteorological stations, located in the districts of Pidu, Wenjiang, Xindu, Shuangliu, Xinjin, and Longquanyi in Chengdu.

**Table 1:** Data of remote sensing and their associated information.

Year	Date of Landsat images	Sensor ID	Landsat entity ID
2010	05-22	Landsat 7 (ETM)	LE71290392010142SGS00
2011	05-25	Landsat 7 (ETM)	LE71290392011145PFS00
2012	09-30	Landsat 7 (ETM)	LE71290392011273PFS00
2013	08-17	Landsat 8 (OLI)	LC81300392013229LGN01
2014	08-13	Landsat 8 (OLI)	LC81290392014225LGN01
2015	04-02	Landsat 7 (ETM)	LE71290392015092EDC00
2016	09-11	Landsat 7 (ETM)	LE71290392016255EDC00S00
2017	05-01	Landsat 8 (OLI)	LC81290392017121LGN00GS00
2018	06-05	Landsat 8 (OLI)	LC81290392018156LGN00T00
2019	08-11	Landsat 8 (OLI)	LC81290392019223LGN00DC01
2020	07-28	Landsat 8 (OLI)	LC81290392020210LGN00DC00

## 3 Methods

**Figure 2:**
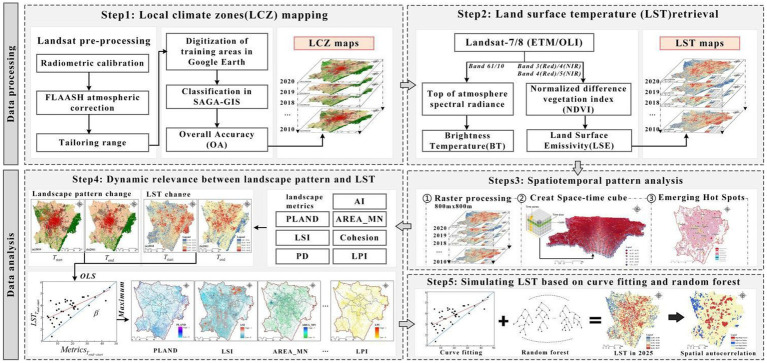
Research and analysis procedures.

The main steps performed are as follows (Figure 2): First, the spatiotemporal evolution of LCZ and LST in study area from 2010 to 2020 was studied, and the relationship between them was analyzed. Second, the LST raster data were reconstructed in 3D, and the STC of LST was used as an input for emerging hotspot analysis to identify the spatiotemporal pattern of LST. Next, the dynamic relationship (DR) between urban spatial pattern changes and LST changes was examined, and the spatial distribution of urban spatial pattern changes with the most significant correlation was revealed. Finally, the model integrating curve-fitting and RF prediction simulated the LST changes in 2025.

### 3.1 Mapping LCZ

**Figure 3:**
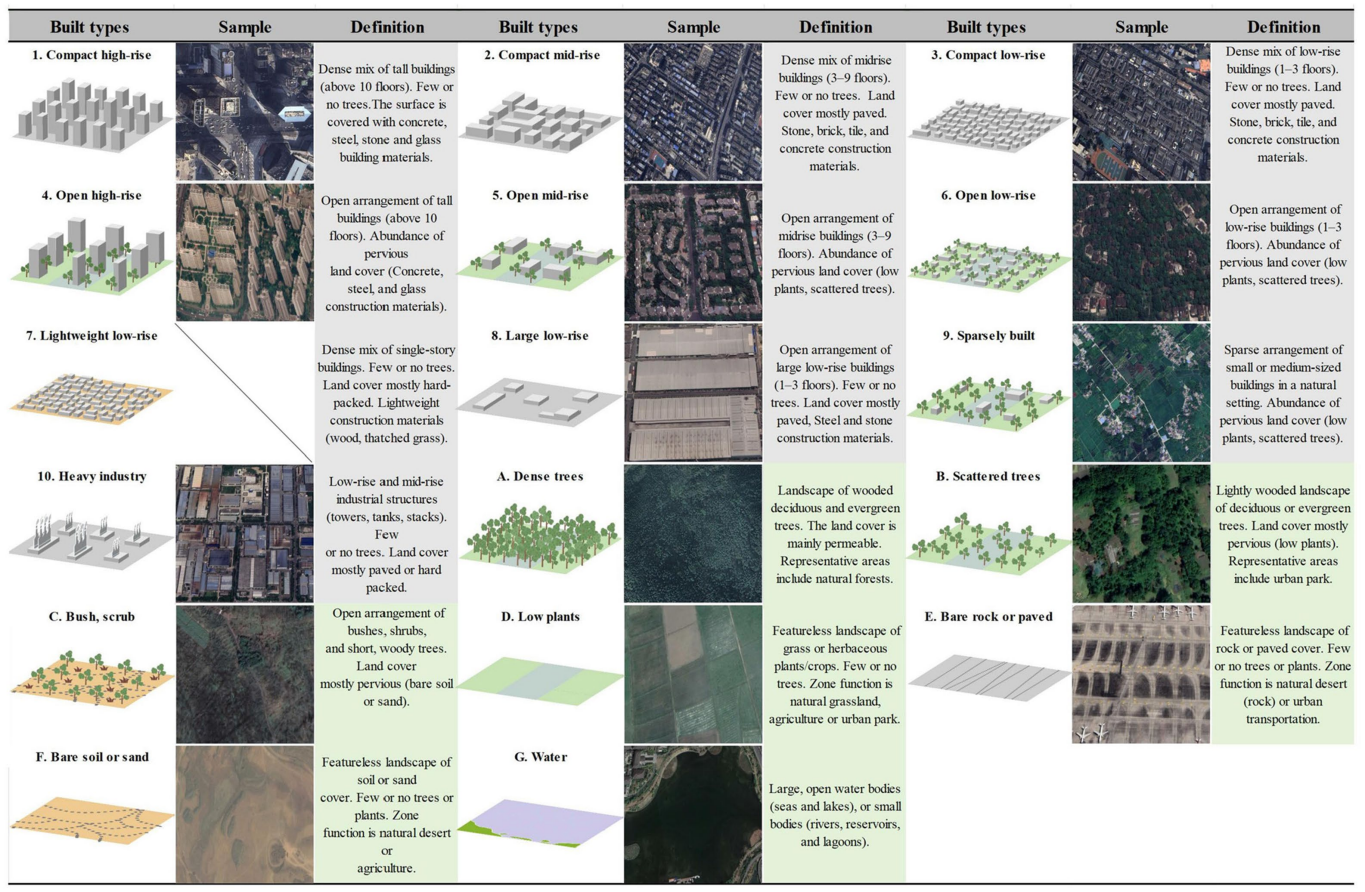
Illustration of LCZ and corresponding image display.

The LCZ classification framework based on the World Urban Database and Access Portal Tools (WUDAPT) was used to identify and categorize urban spatial form. The LCZ system contains 17 distinct categories: 10 built environment types and 7 natural environment types (Figure 3). The steps of LCZ classification are as follows: (1) Image preprocessing: The Landsat image was first preprocessed with radiometric calibration, atmospheric correction, and cropping. Then, resampling was performed using the SAGA GIS platform to adjust the resolution from 30 m to 100 m to obtain the spectral signals of urban features at the local scale (34). (2) Constructing training samples: Based on the historical ultrahigh resolution images on Google Earth Pro, the training zones were digitized using manual visual interpretation; relying on the contemporaneous Baidu Street View maps and field investigation as the basis of collection, each LCZ type contained 30–40 training samples. (3) Classification: The training sample data and preprocessed Landsat satellite spectral data were input into SAGA GIS, and the LCZ were classified using an RF classifier. (4) Accuracy assessment: Four independent groups of validation samples were selected separately on Google Earth Pro, each with about 20 LCZ types. The predicted LCZ were compared with the validation samples to establish a confusion matrix, and the accuracy of the classification was assessed by applying the overall accuracy (OA).

### 3.2 LST retrieval and classification

The radiative transfer equation method was used to retrieve the LST values using the thermal infrared bands of Landsat images. The thermal infrared bands corresponded to bands b1 and 10 of Landsat images 7 and 8, respectively. The specific formulae refer to the study of Sekertekin et al. (35). Several studies have performed for nighttime LST inversion (36) and nighttime light intensity (37). The biggest challenge of this method is to distinguish between nighttime light and thermal anomalies. Therefore we mainly focus on daytime LST. After obtaining the LST data, to eliminate the effect of directly comparing the LST at different times and referring to the previous study on the comparison of the LST division methods (38), the mean–standard deviation method was applied to classify the LST data into different intensities (Table 2).

**Table 2:** Grading standard of urban heat island.

Grades	Detailed zoning	Conditions*
1	High temperature zone (HTZ)	LST > μ + std
2	Sub-high temperature zone (SHTZ)	μ + 0.5 std. < LST < μ + std
3	Medium temperature zone (MTZ)	μ − 0.5 std. < LST < μ + 0.5 std
4	Sub-medium temperature zone (SMTZ)	μ − std. < LST < μ − 0.5 std
5	Low temperature zone (LTZ)	LST < μ − std

*μ: mean LST; std: standard deviation of LST.

### 3.3 STC construction and emerging hotspot analysis for LST

Based on traditional spatial analysis, the STC model and the emerging hotspot analysis were integrated to investigate the spatiotemporal pattern of LST and the spatiotemporal evolution characteristics of LST in each LCZ type. The implementation of this method was joined with ArcGIS Pro, which included the following steps: First, the raster data were processed, and the LST data were added to the mosaic dataset by using “Create mosaic dataset” as a bridge between the multitemporal raster data and the STC data. Next, with the help of the tools “Construct Multidimensional Information” and “Create Multidimensional Raster Layer,” the time dimension variables were constructed in the mosaic dataset and converted into a multidimensional raster layer. Then, using the “Create STC from Multidimensional Raster Layers” tool, the multidimensional raster layers were transformed into STC, which can be considered 3D cubic grid structures with temporal and spatial information (24). Finally, the STC was used as input to monitor the spatiotemporal evolution patterns of the LST using the “emerging hotspot analysis tool,” and Mann–Kendall trend prediction was used to evaluate the hot and cold spots trends of the LST and to obtain the corresponding Z and *p* values (39). If the Z value is greater than 1.65, the time series shows an upward trend, that is, the LST intensity increases from year by year; the opposite also holds true. If the Z value is close to 0, the LST intensity does not change significantly with the change of the time series. The range of the p value determines the level of significance. Based on the score of the Z value and the p value, the LST intensity is categorized into different patterns of hot or cold spots.

### 3.4 Quantification of urban spatial form and its change with LST correlation

#### 3.4.1 Selection of urban landscape pattern indices

Changes in urban landscape patterns reflect the dynamic evolution of urban spatial form in relation to location (40). To quantify the impact of urban spatial forms and their changes on LST, indicators representing landscape composition and configuration were selected from the class-level Fragstats metrics. The criteria for selecting indicators were: to reflect urban form characteristics from multiple perspectives, have practical and theoretical significance, be easy to interpret, and minimize redundancy. Based on previous studies (18, 22, 41), seven landscape indicators were chosen (Table 3): Percentage of Landscape (PLAND) as a composition indicator, Landscape Shape Index (LSI) to represent the complexity of urban landscape shape, Patch Density (PD) to quantify the fragmentation of the urban landscape, Aggregation Index (AI) to indicate the degree of clustering of similar patches in space, Mean Patch Area (AREA_MN) to represent the average size of patches, Cohesion to assess the spatial connectivity of patches, and Largest Patch Index (LPI) to determine the percentage of the largest patch in the landscape.

**Table 3:** Description of landscape metrics used in this paper.

Metric (Abbreviation)	Equation	Description
Percentage of Landscape (PLAND)	$PLAND=P_{i}=\frac{{å}_{j}^{n}=1^{a_{ij}}}{A}\times 100$	Landscape percentage of the corresponding patch.
Landscape Shape Index (LSI)	$LSI=\frac{0.25\times E}{\sqrt{A}}$	Structural composition and spatial configuration within an analysis unit.
Patch Density (PD)	$PD=\frac{{\;}_{n_{i}}}{A}\times 10^{6}$	The density of corresponding patches.
Aggregation Index (AI)	$AI=\left[\frac{{\;}_{g_{ij}}}{\max{(}_{g_{ij}})}\right]\times 100$	Degree of aggregation of the corresponding patches.
Mean Patch Area (AREA_MN)	$\overset{n}{\underset{j=1}{\sum }}\frac{0.25p_{ij}}{\sqrt{a_{ij}}}$	Average shape index of the corresponding patches within an analysis unit.
Cohesion	$\frac{100\left[1-\frac{\sum_{j=1}^{n}P_{ij}}{\sum_{j=1}^{n}P_{ij}\sqrt{a_{ij}}}\right]}{\left[1-{\sqrt{z}}^{-1}\right]}$	Cohesion index measures the physical connectedness of the corresponding patch type.
Largest Patch Index (LPI)	$LPI=\frac{\max\left(a_{ij}\right)}{A}\times 100$	The percentage of the landscape occupied by the largest patch.

#### 3.4.2 Calculation of urban landscape patterns

**Figure 4:**
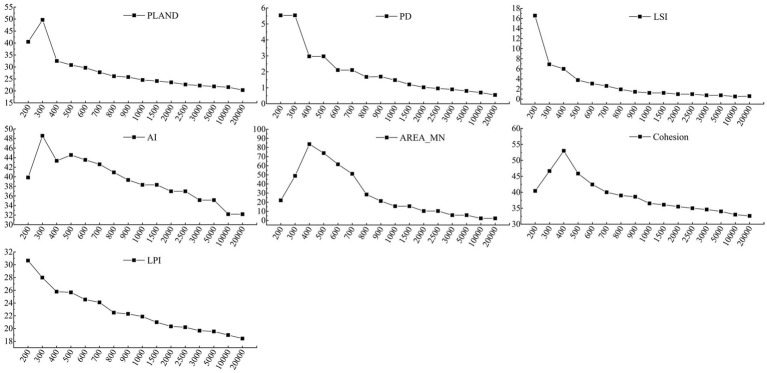
Landscape patterns change of LCZ9 with different window scales.

The scale of the study area and grid size of the raster data will affect the calculation results of the landscape pattern. A grid size of 800 × 800 m was selected. Studies have shown that when the calculation results of the landscape pattern index stabilize with the change of scale, the results are weakened by the influence of scale, and the scale in this trend is considered optimal (42). Before calculating the landscape pattern indices, the changes in landscape patterns were analyzed using the moving window method. Landscape changes at 16 different scales (n = 200, 300, 400, 500, 600, 700, 800, 900, 1,000, 1,500, 2000, 2,500, 3,000, 5,000, 10,000, 20,000 m) at the landscape level were calculated, and then the largest area, most widely distributed built-up landscape type (LCZ9) in the study area was selected to calculate the changes in its landscape indices at different scales. As shown in Figure 4, with the increase in distance, the trend of change in landscape pattern indices gradually decreases after 800 m, indicating that the landscape pattern indices are less sensitive above 800 m.

#### 3.4.3 Dynamic correlation between changes in urban spatial patterns and changes in LST

Assessing the relationship between changes in urban spatial patterns and LST is of great significance for emphasizing spatial considerations in urban climate mitigation studies. Ordinary Least Squares (OLS) modeling estimates the parameters in a regression model by minimizing the sum of squared residuals. In our study, a correspondence between changes in urban spatial form and changes in LST was assumed, and considering geographically weighted regression models will cause collinearity problems, the OLS model was chosen to calculate the regression relationship between changes in urban spatial patterns and changes in LST as follows (Equation 1):


(1) $$Y_{ij}^{LST}=\beta X_{ij}^{Metrics}+\varepsilon ,$$

where $Y_{ij}^{LST}$ is the LST of grid ij; $X_{ij}^{Metrics}$is the metric of urban spatial pattern to the corresponding grid of $Y_{ij}^{LST}$; β is the regression coefficient of the change in the urban spatial pattern that is fitted by using the OLS principle of choosing the parameters of the linear function of the explanatory variables, which reflects the change in the urban spatial pattern on the degree of influence of LST. Dynamically, the correspondence between urban spatial pattern and LST evolved into the correspondence between urban spatial pattern changes and LST changes in this paper. The independent variable is the difference in spatial pattern measurement of the city where the grid is located between the end time and the start time, and the dependent variable is the LST difference of the corresponding grid between the end time and the start time as follows (Equation 2):


(2) $$Y_{ij}^{LST_{Tend}}-Y_{ij}^{LST_{Tstart}}=\beta \left(X_{ij}^{Metrics_{Tend}}-X_{ij}^{Metrics_{Tstart}}\right)+\varepsilon$$

Prior to the OLS regression analysis, the values of each measure of the independent variables were normalized to eliminate the effect of differences due to unit inconsistencies as follows (Equation 3):


(3) $$N_{ij}^{Metrics}=\frac{X_{ij}^{Metrics}-X_{\min}^{Metrics}}{X_{\max}^{Metrics}-X_{\min}^{Metrics}}$$

On this basis, to reflect the spatial characteristics of urban spatial pattern changes significantly correlated with the LST rise from 2010 to 2020, the type of LCZ with the most significant correlation between urban spatial pattern changes and increases in LST in each of the selected seven landscape indices across the years were identified. Next, the regression coefficients of LCZ changes and LST over the 10-year period were cumulatively summed to obtain the change in the rank intensity of urban spatial patterns affected by the LST rise, which was normalized for comparison and analysis due to the enormous difference in the values of the pooled regression coefficients.

### 3.5 LST simulation based on curve-fitting and RF prediction

The results of multiple forecasting models were considered, and the best forecast for each location was selected. The dynamic changes in LST were incorporated into the forecasting utilized LST data from 2010 to 2020, and a combination of a linear regression model based on curve-fitting and a nonlinear machine learning model based on RF was used to obtain the simulation results of the 2025 LST by evaluating the accuracy of the results of these two models by location. Spatiotemporal pattern mining based on the RF algorithm was used to train the RF regression model by using the time window at each location of the STC. Spatiotemporal pattern mining based on curve fitting involves fitting a parametric curve to each position in the input STC parameters and predicting the time series by extrapolating this curve to future time steps. Each prediction model was constructed for two purposes: a prediction model for predicting future time step values and a validation model for validating the prediction model and evaluating the accuracy of its predicted values. Evaluation by location can compare and merge multiple predicted STC cubes and uses the “Root Mean Square Error (RMSE) of Prediction” or “Validation RMSE” values to identify the best prediction for each location.

## 4 Result and analysis

### 4.1 Analysis of changes in urban spatial form

#### 4.1.1 Accuracy check and overall analysis

**Figure 5:**
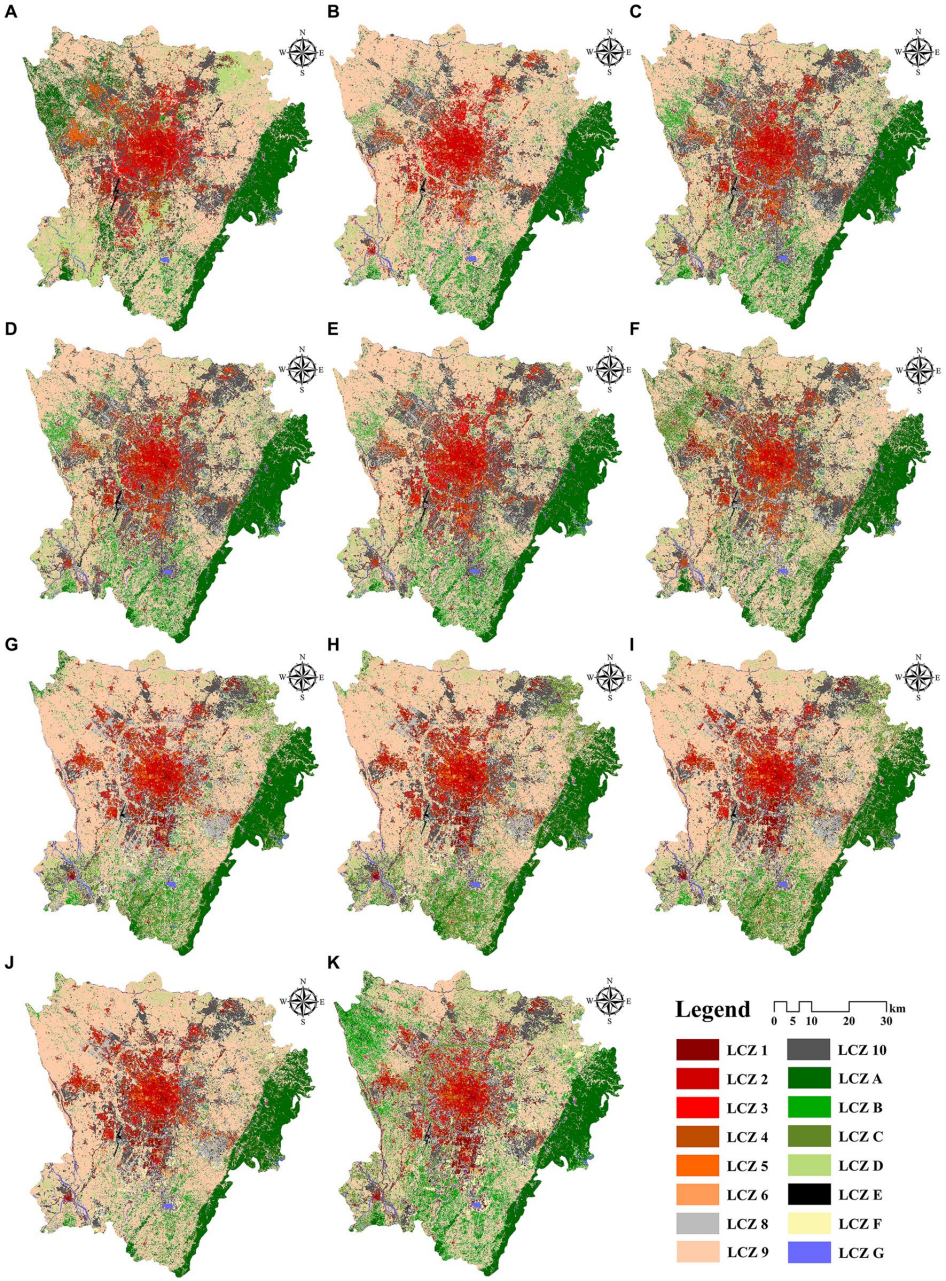
LCZ maps obtained for: **(A)** 2010–**(K)** 2020.

Figure 5 illustrates the LCZ classification results for study area from 2010 to 2020. LCZ maps through the WUDPT portal should ensure at least 50% accuracy (43), and the accuracy of existing LCZ maps in China is generally higher than 60% (44). In the end, the OA of LCZ for each year in our study reached over 65%, and for types with simple, easily recognizable forms such as LCZ2, LCZA, and LCZG, the accuracy exceeded 80%. The original LCZ framework contained 17 types, but due to the lack of LCZ7 in Chengdu, the final classification result includes 16 LCZ types. Changes in the spatial distribution of LCZ show the spatial pattern of the city has changed significantly over the past decade, especially in the southeast, where rapid urban growth has been experienced. The area of LCZ9 is large because many western Sichuan Linpans are spread around the Chengdu central district, which is consistent with the urban form of sparse buildings distributed in the natural environment. The urban form of study area has been dominated by the widely distributed LCZ9, followed by LCZ10 (Table 4). With the change of urbanization and land use patterns, the subdominant form gradually transformed from LCZ3 in 2010 to LCZ1 in 2020. LCZA and LCZD declined trend to 2020 reverse. The area of LCZE, represented by rock and hard paving, is rising.

**Table 4:** Share of the LCZ types in study area from 2010 to 2020 (km^2^).

Year	LCZ1	LCZ2	LCZ3	LCZ4	LCZ5	LCZ6	LCZ8	LCZ9	LCZ10	LCZA	LCZB	LCZC	LCZD	LCZE	LCZF	LCZG
2010	32.39	171.02	200.49	68.80	98.44	80.17	58.42	1659.18	423.37	666.17	81.97	40.59	332.75	24.65	31.66	36.88
2011	59.37	177.01	119.64	50.74	74.14	73.68	44.79	2225.07	307.82	454.52	161.83	52.08	99.10	18.388	30.40	58.67
2012	57.10	124.15	82.01	129.30	57.06	109.11	133.18	1713.01	636.13	429.61	229.54	61.87	124.67	20.14	45.86	54.20
2013	64.42	126.68	78.01	128.10	54.33	113.34	126.66	1689.88	634.68	433.35	237.75	60.69	126.54	33.38	43.54	55.87
2014	65.56	107.80	156.64	112.62	51.17	91.86	115.96	1738.66	593.52	433.16	229.72	58.43	122.35	31.44	44.86	53.48
2015	89.01	82.77	30.87	131.45	75.22	177.48	128.60	1636.22	592.37	527.90	91.11	82.41	201.50	16.12	88.35	55.84
2016	92.60	189.03	28.37	136.49	30.02	114.47	297.00	1731.37	302.92	403.55	140.98	226.46	138.13	12.22	101.07	62.67
2017	111.45	148.78	43.92	117.78	43.47	131.73	199.85	1764.24	400.10	374.29	95.47	267.61	132.68	18.41	106.61	50.84
2018	139.50	160.13	42.23	96.77	39.12	138.90	256.95	1798.42	371.06	373.91	101.34	227.61	131.02	14.40	68.67	47.44
2019	137.46	161.22	46.19	93.82	35.27	127.79	208.35	2031.59	364.84	366.01	65.84	116.67	85.75	20.34	100.73	45.35
2020	182.58	112.12	62.19	80.24	38.09	133.51	170.39	1454.35	330.52	445.02	411.86	220.26	109.33	41.98	163.09	51.70

#### 4.1.2 Analysis of urban spatial form transfer

**Figure 6:**
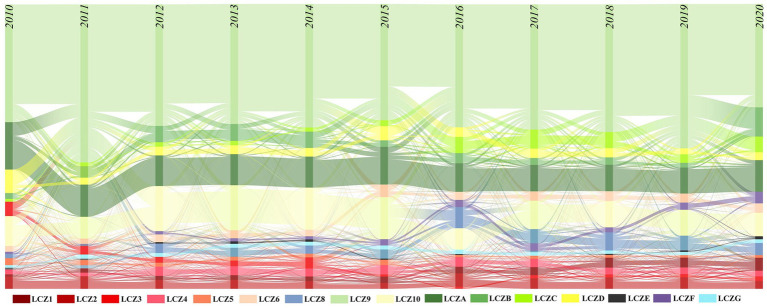
Transfer results of LCZ types from 2010 to 2020.

The changes in urban spatial form reflect the development process of the city (Figure 6), which is generally divided into three stages: (1) Incremental expansion stages from 2010 to 2013. The city experienced the initial stages of growth, showing a transition from a natural environment to a built environment dominated by sparse low-rise forms. Urban sprawl and the rapid increase of low-density buildings in the urban fringe areas. The expansion of LCZ9 converted from LCZA was the main landscape change in this phase. (2) Coexistence of incremental expansion and stock renewal stages from 2014 to 2016. The natural environment continued to transform into the built environment, while urban buildings began to develop in the direction of densification and verticalization. Especially in the construction of new urban areas such as Tianfu New District, many high-rise buildings were built one after another, changing the original pattern of low-rise buildings in Chengdu. The increase in the area of dense LCZ was mainly due to the conversion from other built-up types. For example, the area of LCZ1 doubled six times during this period, mainly from the conversion of LCZ2-5. (3) Coexistence of stock renewal and the ecological transformation phase from 2017 to 2020. Urban functions were continuously upgraded, and the built-up types of LCZ shifted to mid- and high-rise forms that can intensively land use; at the same time, the transfer of the natural environment to the built-up environment slowed down, indicating Chengdu’s urban planning increasingly emphasized environmental protection and ecological construction.

#### 4.1.3 Analysis of changes in urban spatial pattern

**Figure 7:**
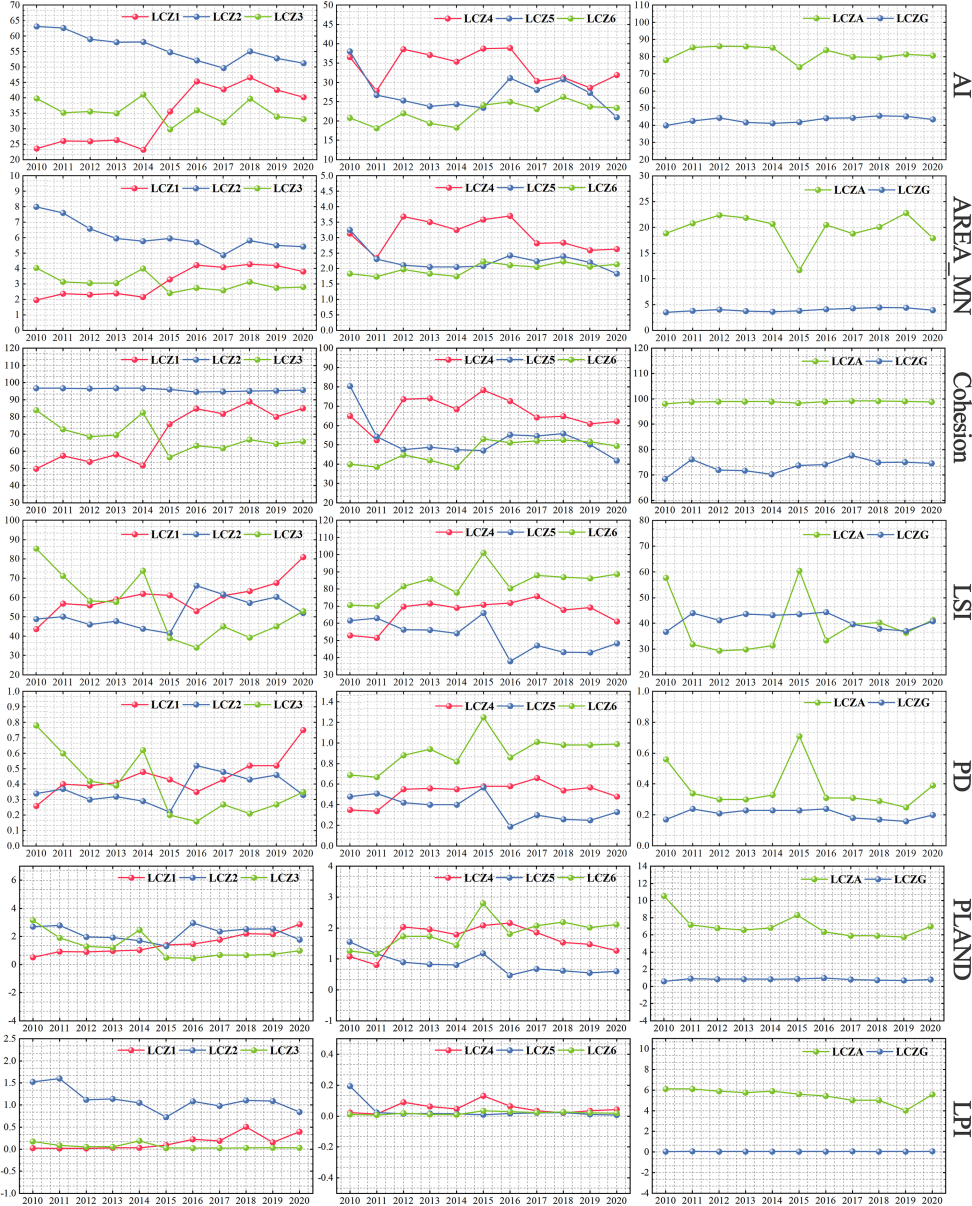
Changes in urban spatial pattern from 2010 to 2020.

In this study, we selected LCZ1-6, LCZA, and LCZG from 16 LCZ types for the analysis of landscape pattern changes. LCZ1-6 represent different levels of building density and height within urban areas, reflecting variations in population density, economic development, and urbanization levels. LCZA and LCZG typically occur in natural areas with better ecological environments. These LCZ types allow for homogeneous comparisons of building density or height as well as comparisons between built environments and natural settings. Changes in landscape patterns were diverse due to differences in the amount of urban form and spatial layout (Figure 7). Overall, the changes in AI, AREA_MN, and Cohesion; LSI, PD, and PLAND generally follow similar trends, while the changes in PLAND and LPI are relatively minor. Change in the landscape pattern of dense built-up environments was greater than that of open built-up environments, and the change in the landscape pattern of water was smaller. In terms of the dense built environment, the landscape pattern indices of LCZ1 all showed an upward trend, and 2014–2016 was the stage of dense, vertical development of urban spatial form. Therefore, the AI, AREA_MN and Cohesion of LCZ1 increased significantly, which was about twice as much as that of the previous one. The landscape patterns of LCZ2 and LCZ3 overall show a declining trend. The changes in openly built environments did not show a clear trend throughout the time scale and mainly fluctuated between years. The AI, AREA_MN and Cohesion of LCZ4 led LCZ5 and LCZ6, with LSI, PD and PLAND in between. In terms of nature environments, the AREA_MN and AI of LCZA fluctuated significantly during 2014–2016 and changed in the opposite direction to its LSI, PD and PLAND. Between 2014 and 2015, the area of LCZA increased, but AI and AREA_MN decreased while LSI, PD and PLAND increased. This may be due to the newly added green spaces in the city being relatively small and dispersed. Between 2015 and 2016, the area of LCZA decreased, but AI and AREA_MN increased, and LSI, PD and PLAND decreased, which is due to the reduction in fragmented green spaces, thereby increasing the continuity and integrity of the landscape.

### 4.2 Changes and analyses of urban thermal environment based on LCZ

#### 4.2.1 Overall analysis of changes in urban thermal environment

**Figure 8:**
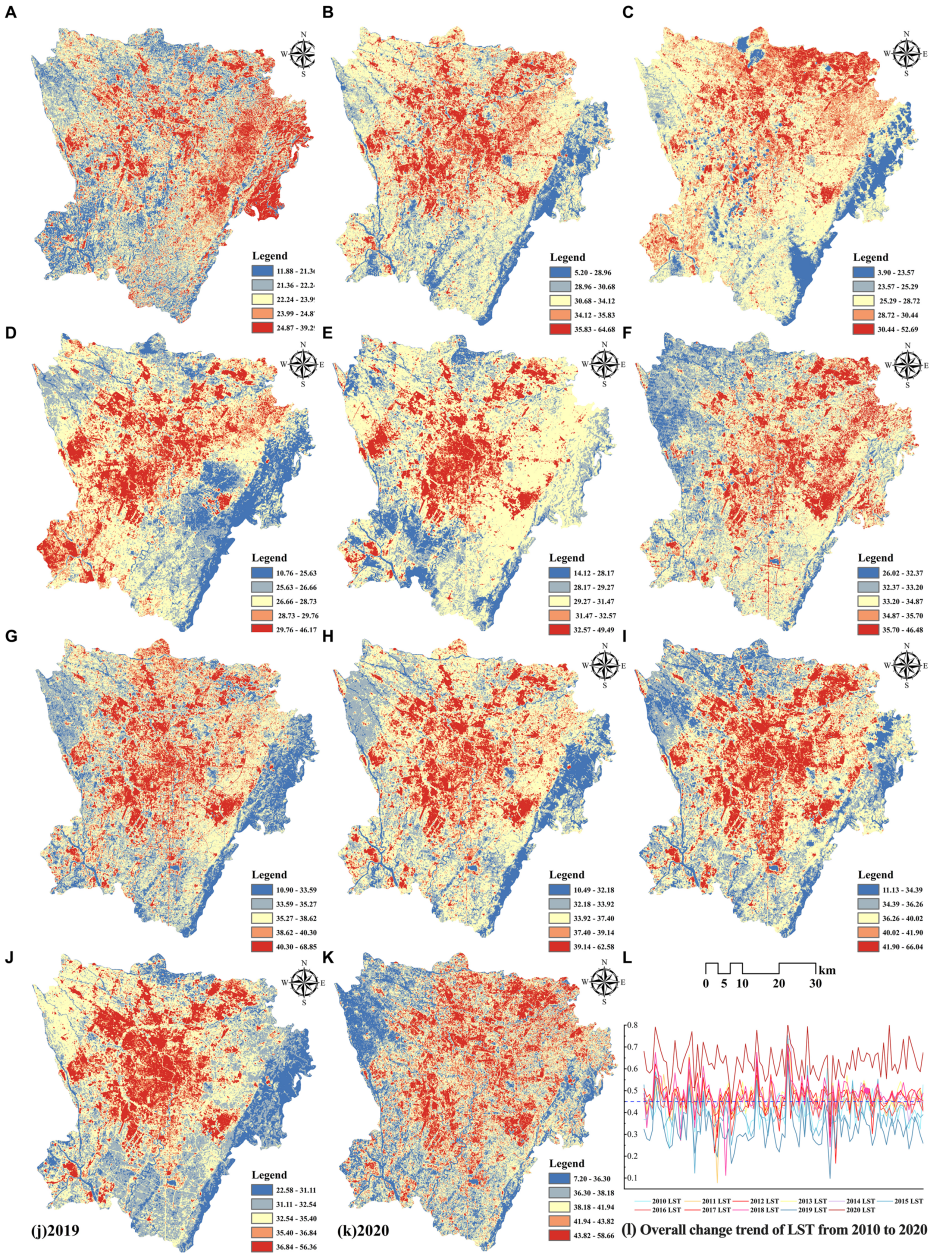
LST distribution for: **(A)** 2010–**(K)** 2020; **(L)** overall change trend of LST.

Figure 8 shows the spatial distribution of the urban thermal environment in study area during 2010–2020 as quantified by the LST pixel histogram. The results show spatial differences in the thermal environment in the past decade, and the high-temperature zone exhibited a radial distribution from the city center to the surrounding areas. Except for 2012, when the spatial distribution of the thermal environment was more anomalous, all the other years showed heat island centers and peripheral cold island zones. In 2010, the Longquan mountain range area in the southeast of Chengdu showed a high LST. The remote sensing imagery revealed a large amount of exposed reddish-brown soil in the area, and the vegetation cover was low, which resulted in a high LST. To reveal the overall trend of LST changes in the past decade, the LST normalization results for 100 random sample points in the study area from 2010 to 2020 were obtained (Figure 8L). The LST in Chengdu showed an overall warming trend. Despite the differences in the LST values retrieved in different years, the trends of the average LST changes in the same geographic location under different years were the same, indicating the distributions of the urban heat field in the same region in different years are consistent on a large scale while fluctuating significantly in a small range. The average LST from 2010 to 2020 were 23.12, 26.40, 27.01, 27.69, 30.37, 34.03, 36.95, 35.66, 38.14, 33.97, and 40.06°C. Corresponding air temperatures were 22.22, 24.18, 24.35, 28.70, 25.27, 28.82, 31.98, 31.70, 34.65, 30.87, and 36.62°C, respectively. All differences were within the normal range, and further research can be conducted.

#### 4.2.2 Comparative analysis of LST for each LCZ

**Figure 9:**
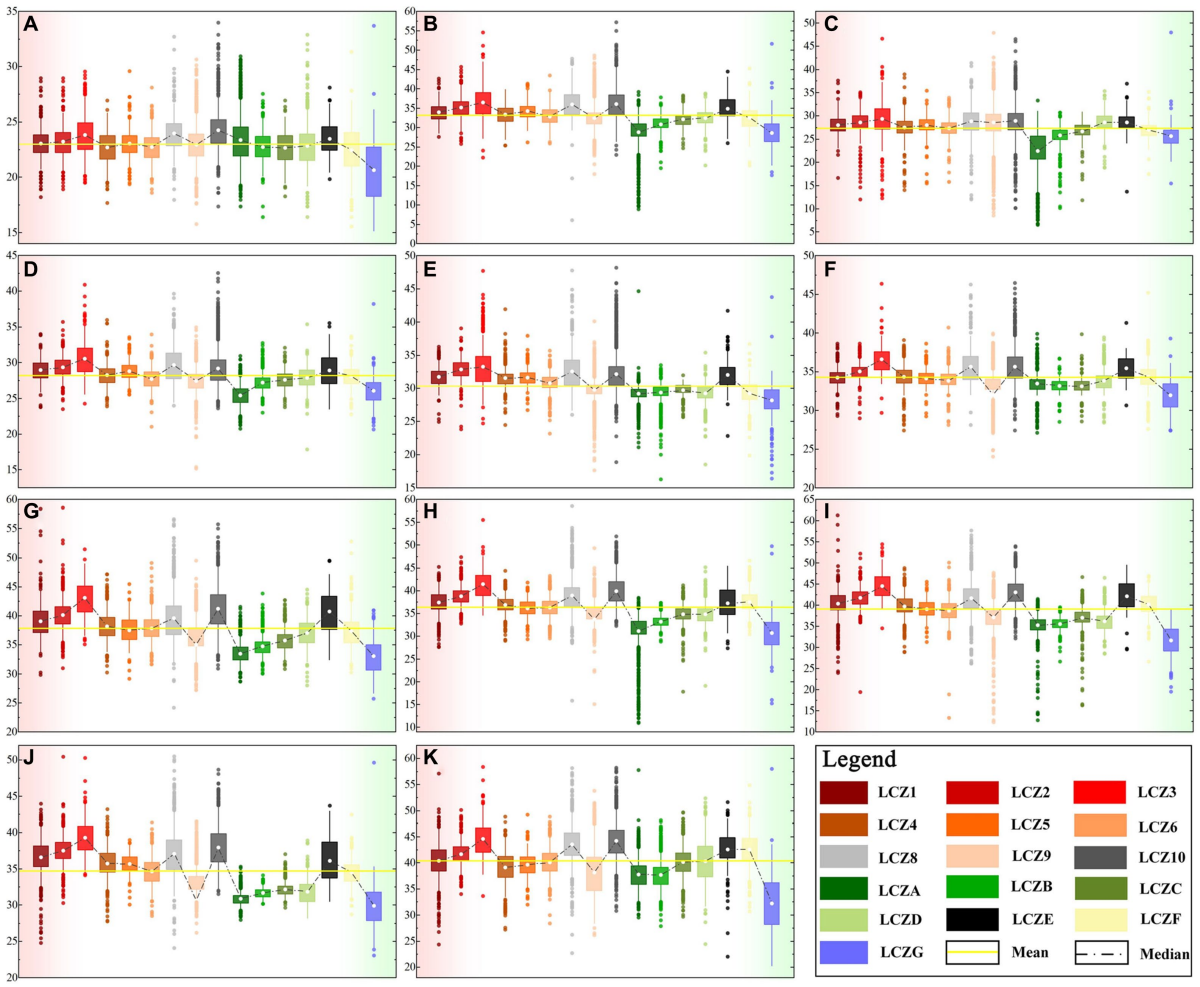
Box plots of LST in different LCZ types from: **(A)** 2010–**(K)** 2020.

Figure 9 shows the LST of each LCZ from 2010 to 2020. Although the LST of each LCZ type represented different periods, the LCZ and LST data came from the same remote sensing image, and some general trends can be observed. The LST of built-environmental LCZ is generally higher than that of natural-environmental LCZ. LCZ6 is the closest to the average LST. LCZG always has a lower LST than the average LST. The LST of compact LCZ is higher than that of open LCZ with comparable building heights, that is, LCZ1 > LCZ4, LCZ2 > LCZ5, and LCZ3 > LCZ6, because compact LCZ have a high density of hard paving and fewer spaces for ventilation and cooling at the same building heights. Among dense buildings, the LST of low-rise LCZ is higher than that of high-rise LCZ, for example, LCZ3 > LCZ2 > LCZ1, because high-rise buildings can play the role of shade between floors. Among open buildings, the LST of low-rise LCZ is lower than that of high-rise LCZ, that is, LCZ6 < LCZ5 < LCZ4, which is different from that of dense buildings in that open buildings cannot play the role of shade between floors efficiently, and under a certain degree of openness case, low-rise buildings contain less surface heat capacity and warm up slowly after absorbing heat, so the LST is lower. LCZ10 has the higher LST in the built environment due to the emission of heat sources and higher industrial albedo. For natural environments, LCZA and LCZG have the lowest LST. Among trees of different heights and densities, the mitigation of the LST effect presents LCZA > LCZB > LCZC, and the effect of other natural types, such as LCZD and LCZF, in mitigating LST is moderate. The LST of LCZE is prominent and even higher than that of some built-up LCZ.

#### 4.2.3 Analysis of LST spatiotemporal patterns based on the STC model

**Figure 10:**
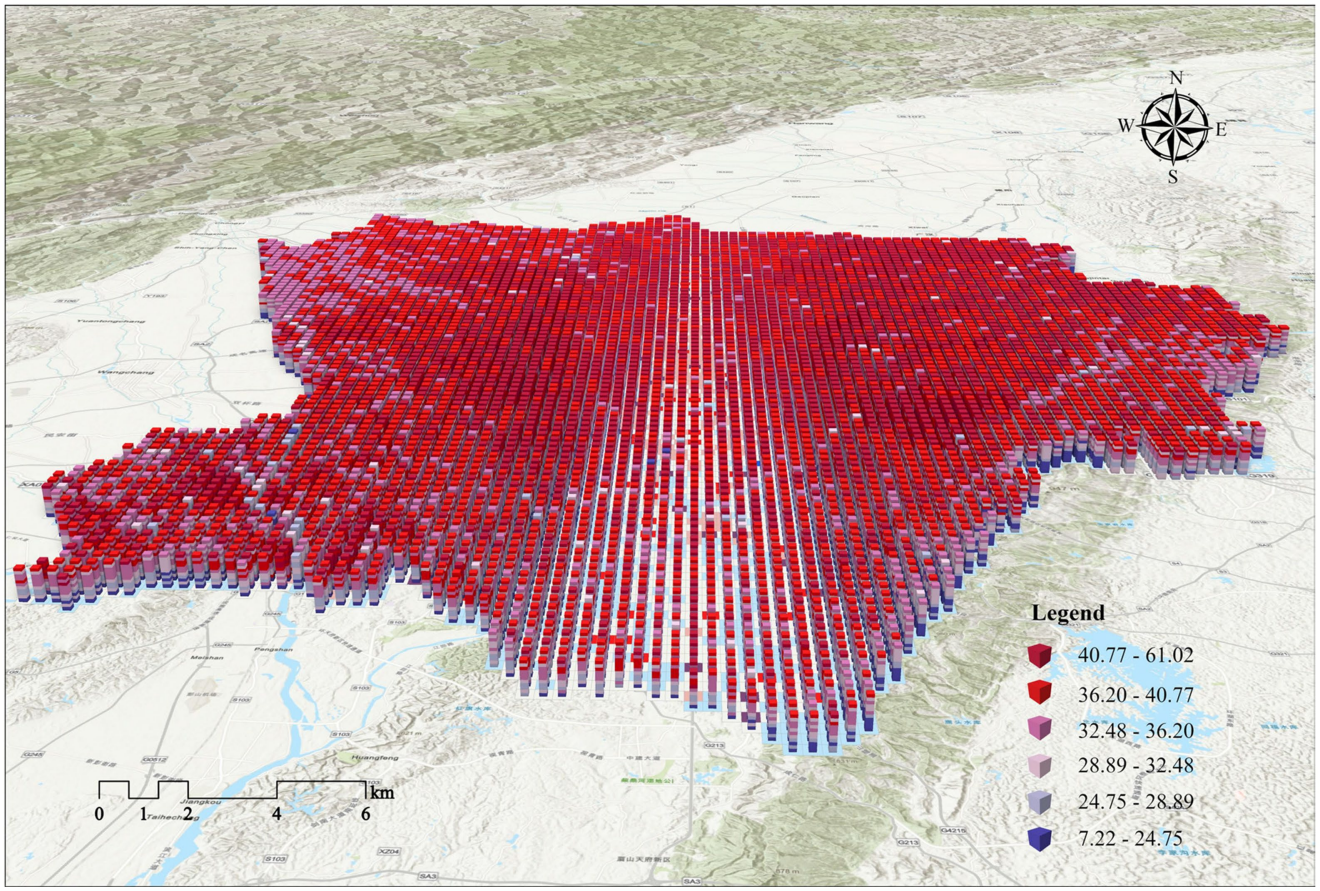
STC of LST from 2010 to 2020.

**Figure 11:**
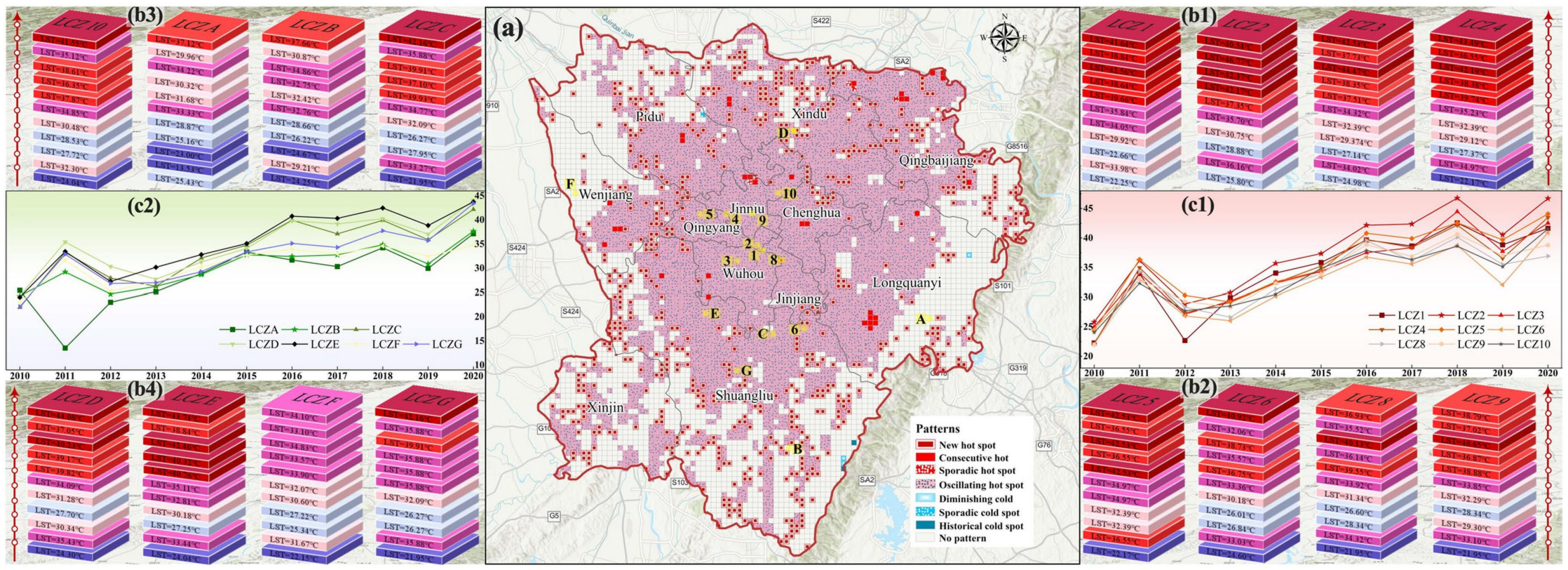
**(a)** Spatiotemporal patterns of LST from 2010 to 2020; **(b1-b4)** STC of LST changes of each LCZ; **(c1-c2)** line chart of LST changes of each LCZ.

The LST raster data from 2010 to 2020 were reconstructed in 3D using annual LST timestamp information, and Figure 10 illustrates the STC of the LST data. Figure 11a shows the results of the emerging hotspot analysis of LST spatiotemporal patterns. Seven spatiotemporal patterns of LST were identified in Chengdu: consecutive, new, oscillating, and sporadic hotspots; diminishing, historical and sporadic cold spots. Oscillating hotspots accounted for the largest proportion in the study area, namely, 51.99%, followed by new hotspots with a proportion of 11.44%. Oscillating hotspots patterns were most significant in Jinniu, Chenghua, Qingyang, Wuhou, and Jinjiang districts, indicating these districts had significant hotspots in the entire time frame and significant cold spots in the previous time frame. New hotspots have recently emerged, which are mainly distributed in the areas of peri urban LCZ9 as a result of urban expansion. Consecutive hotspots are areas that remain hot throughout the study period and are mainly distributed in the areas of LCZ2, LCZ3, and LCZ10. The periphery of study area had sporadic distributions of diminishing, historical, and sporadic cold spots, which were mainly distributed in areas of stable vegetation cover. The northwestern and southeastern areas, where the thermal environment characteristics is not significant due to natural geographic conditions, are considered areas where the pattern is not monitored. This paper reveals the spatiotemporal variation patterns of LST intensity based on its temporal and spatial variations over the past 10 years and identifies the main hotspots patterns in Chengdu: oscillating hotspots > new hotspots (less than 1% of consecutive and sporadic hotspots).

To reveal the spatiotemporal changes in LST for each LCZ type, we extracted the urban spatial forms that remained unchanged from 2010 to 2020 (specifically, the centroids of intersecting patches of each LCZ over the decade) and analyzed the variations in their LST during this period. Figures 11b1–b4 show the STC of the LST of each LCZ from 2010 to 2020, with a spatial scale of 800 × 800 m. The LST of LCZ in the built environment and the natural environment showed an upward trend (Figures 11c1,c2). Among the densely built environments, LCZ2 had the greatest upward trend in LST. In openly built environments, LCZ4 had the largest upward trend in LST. The rise in LST in LCZ9 in the built environment was also quite significant. Among the natural types, LCZC showed a more pronounced LST rise relative to LCZA and LCZB. In addition, the warming trends of LCZD and LCZE were significant. This analysis method is conducive to observing differences in the intensity of LST changes between the different LCZ as well as spatiotemporal trends in LSTs within each LCZ.

### 4.3 Relevance between urban spatial pattern changes and LST changes in dynamic way

**Figure 12:**
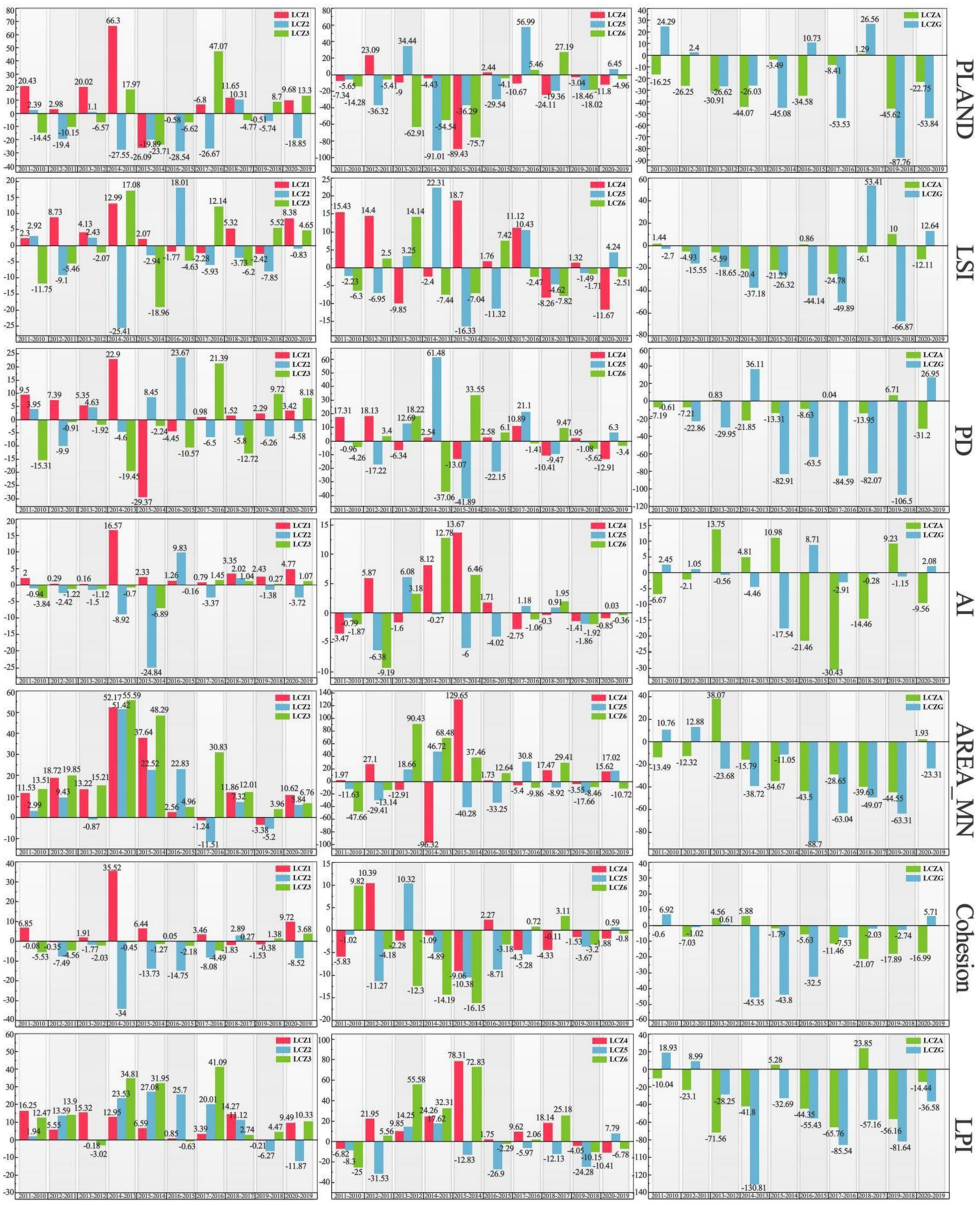
Dynamic regression between urban spatial pattern and LST.

Figure 12 presents the DR between changes in urban spatial patterns and changes in LST. The influence of different time periods and types of built environments on LST varies, while the influence of natural environments on LST is always negatively correlated. Changes in AREA_MN and PLAND have a greater effect on LST than changes in AI and Cohesion. In determining the DR, the effect of time series was comprehensively considered. The DR between changes in landscape pattern and changes in LST between adjacent years for each LCZ was analyzed, and more than half of the positive and negative correlations as the final DR were selected. The analyses of the relationship between changes in each landscape index and change in LST reveal the following:

According to PLAND, LSI, and PD analysis, in densely built environments, changes in LCZ1 are positively correlated with LST, whereas LCZ2 and LCZ3 are negatively correlated. This indicates that increases in PLAND, LSI, and PD in dense high-rise buildings lead to higher LST, whereas similar increases in mid and low-rise buildings have a smaller effects on UHIE. High-rise buildings, which typically have more heat-absorbing surfaces and complex structures and shapes, therefore have a greater effects on UHIE. In the open-built environment, changes in the LSI have a similar effects on LST as in dense built environments. This finding indicates the effects of LSI on LST is related to the height of the building, with some consistency across different building densities. The changes in PD for LCZ4 and LCZ6 are positively correlated with LST, while for LCZ5, the correlation is negative. Changes in PLAND are negatively correlated with LST across these zones, indicating that compared to open mid-rise buildings, the increase in high and low-rise buildings has a greater impact on UHIE. However, increasing the proportion of permeable or low heat capacity ground cover helps alleviate UHIE. In natural environments, changes in PLAND, LSI, and PD for LCZA and LCZG are negatively correlated with LST, demonstrating that increasing the proportion of vegetation and water, as well as the density and complexity of patches, can effectively mitigate the UHIE.

According to the analysis of AI and Cohesion, changes in LCZ1 are positively correlated with LST, whereas changes in LCZ2 and LCZ3 show a negative correlation. This occurs because in dense high-rise buildings, increased clustering and connectivity lead to narrow gaps between buildings, creating “urban canyons” that restrict air circulation and facilitate the accumulation of heat. In contrast, changes in AI and Cohesion in open-type buildings are negatively correlated with LST. The degree of dispersion of buildings is greater in the open-built environment, and this dispersed structure helps provide more ventilation and air circulation, along with a higher level of greenery than in dense buildings. In natural environments, changes in LCZA are negatively correlated with LST, benefiting from the transpiration and shading provided by trees, which help reduce LST. The changes in LCZG are negatively correlated with LST. Due to the high specific heat capacity of water, they absorb and store heat during the day and slowly release it at night, effectively regulating LST.

According to AREA_MN and LPI analysis, changes in built-up environments are mostly positively correlated with LST, and changes in natural environments are all negatively correlated with LST. The effect of changes in dense built environments on the rise in LST shows a relationship of LCZ3 > LCZ1 > LCZ2. Low-rise buildings have larger footprints and less shading between buildings, and the changes in AREA_MN and LPI have a more significant effect on LST. High-rise buildings are characterized by floor-to-floor shading, which reduces some of the heat absorption. By contrast, midrise buildings are lower in height, which is more conducive to air circulation and has less influence on UHIE. In the open-built environment, the effect on LST increase is shown as LCZ4 > LCZ6 > LCZ5. LCZ4 has a higher building height and contains more hard surfaces, and LCZ6 has a larger footprint, and their changes have a greater effect on UHIE. By contrast, midrise buildings are more conducive to reducing the rise in LST due to solar radiation. In natural environments, changes in LCZA and LCZG show negative correlations with LST, indicating larger areas of vegetation cover and water are more conducive to mitigating UHIE.

### 4.4 Urban spatial patterns closely associated with LST rise

**Figure 13:**
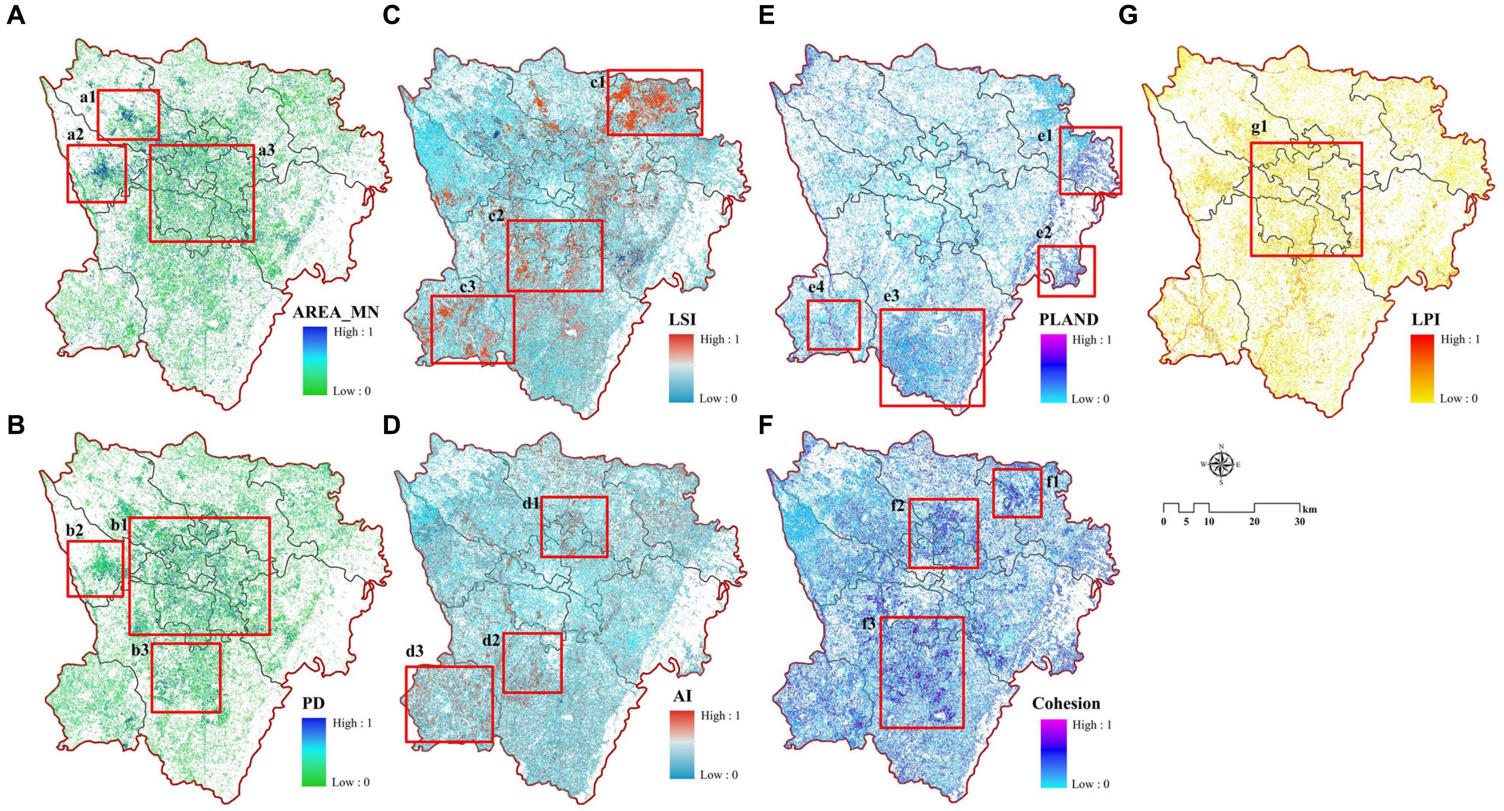
Relationship between landscape pattern and LST rise: **(A)** AREA_MN; **(B)** PD; **(C)** LSI; **(D)** AI; **(E)** PLAND; **(F)** Cohesion and **(G)** LPI.

Figure 13 show the spatial distribution of landscapes, where different landscape pattern indices are strongly associated with LST rise during 2010–2020. Spatially, the changes in AREA_MN and PD that influence LST have similar distribution characteristics, primarily concentrated in urban centers, with relatively weaker correlations in the urban peripheries (Figures 13A,B). Although AREA_MN has the greatest influence on LST, the landscape area of LST rise associated with AREA_MN is relatively small because the changes in urban spatial form affected by AREA_MN are mainly the built-up LCZ in the city, where changes in building area and density significantly influence the rise in LST. Changes in LSI, AI, Cohesion, and PLAND are more widespread and evenly distributed spatially (Figures 13C–F), with LSI showing significant small-scale clustering in the northeastern part of the city (Figure 13c1), indicating that changes in landscape shape complexity in this area have a substantial impact on LST. Changes in PLAND that significantly affect the rise in LST are distributed in the southeastern Longquan Mountain area, and the spatial distribution of LPI influence on LST is the smallest (Figure 13G). In terms of area, the landscape metrics influencing LST from largest to smallest are Cohesion, LSI, AI, PLAND, AREA_MN, PD, and LPI, respectively covering 2355.89 km^2^, 2242.53 km^2^, 1984.99 km^2^, 1369.85 km^2^, 909.44 km^2^, 887.81 km^2^, and 837.74 km^2^, accounting for 58.80, 55.96, 49.53, 34.19, 22.70, 22.15, and 20.91% of the total study area, respectively.

### 4.5 LST feature analysis based on curve fitting and RF prediction

**Figure 14:**
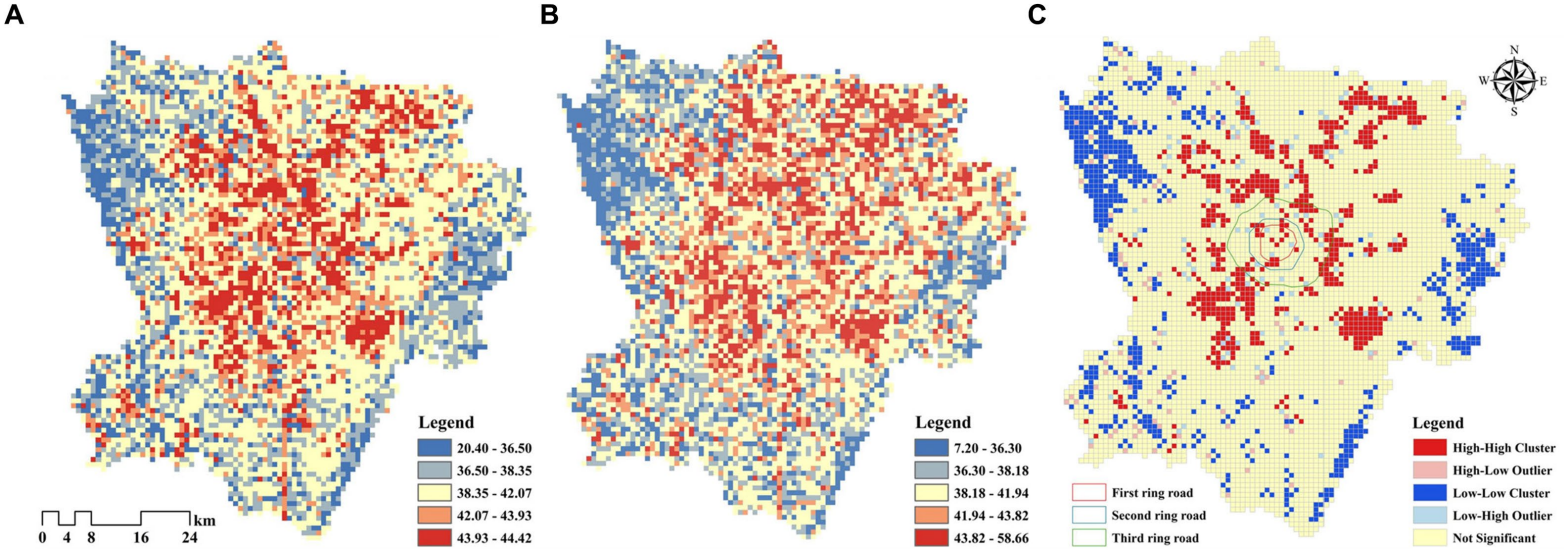
**(A)** Simulation results of LST in 2025; **(B)** LST in 2020; **(C)** Local autocorrelation characteristics of LST in 2025.

Figure 14A illustrates the LST simulation results for study area in 2025. The model shows a prediction RMSE of 0.048 and a validation RMSE of 0.053. Our RMSE value is lower than previous LST prediction studies with a single model (RMSE = 0.059) (41), which shows the multi-model integrated prediction method can effectively present the future structure of the urban thermal environment. Compared with the LST in 2020 (Figure 14B), significant changes are as follows: Quantitatively, the simulated thermal environment in 2025 has an average LST of 40.21°C, which is expected to increase by 0.15°C compared with 2020. Spatially, first, the conversion of low- and sub medium-temperature zones into medium temperature zones with higher LST in most areas, and the significant increase in medium-temperature zones implies the overall thermal environment of the city will be generally warmer. Second, the extent of high and sub high temperature zones are concentrated in urban centers, and the extent of UHIE-affected areas will become more clustered in urban cores in the future. The results of the global spatial autocorrelation analysis (Figure 14C) show that the Moran’s I is 0.40, which is significant at the 5% level. This indicates that there is a significant spatial autocorrelation in the urban thermal environment overall in the future. The high-temperature zones form “high–high” clusters, continuing the thermal characteristics from 2020. These clusters are mostly distributed outside the Third Ring Road of Chengdu. The “low–low” clusters are in Wenjiang District in the north-west, Xinjin District in the south-west, and Longquan Mountain Range in the east of the study area.

### 4.6 Discussion

#### 4.6.1 Enhancement of research on the relationship between the evolution of refined urban spatial patterns and the thermal environment

The previous single LULC classification system ignored the complexity and spatial heterogeneity of the urban landscape structure (45), such as the variation of building heights and densities within the city and the differences in vegetation cover in the natural environment. Fine-grained evolutionary analyses of urban spatial patterns can help reveal the transitions of different properties within the built and natural environments of cities as well as the patterns of transitions between the natural and built environments of cities. Such observations provide an important basis for understanding the influence of urban spatial form on the urban thermal environment at the regional scale. The spatial correlation and heterogeneity of urban spatial form and LST are often easily overlooked in urban climate mitigation strategies. Previous one-size-fits-all design strategies have failed to reflect the thermal mitigation capacity of land surface properties accurately (46), and spatial inequalities in climate change mitigation may occur. Therefore, identifying urban spatial patterns closely related to LST rise under refined LULC classification helps planners and decision makers develop urban planning and thermal mitigation strategies according to the specific conditions of the city.

#### 4.6.2 Examining the influence of changing urban landscape patterns on LST in a dynamic way

Changes in urban landscape patterns are one of the crucial factors in the formation of UHIE. Previous studies have revealed the static correlation between landscape pattern and LST (18, 47), that is, the relationship between landscape pattern and LST at a specific time, which only reflects the static influence of landscape pattern on LST. However, the urban spatial form is constantly changing, and the change in landscape pattern has a dynamic effect on LST, which is an evolving characteristic that cannot be captured by static studies. Therefore, the relationship between changes in urban landscape patterns and changes in LST were examined from a dynamic perspective. Specifically, the study focused on the relationship between the difference in the independent variable and the difference in the dependent variable to reveal how changes in landscape patterns affect changes in the urban thermal environment. In addition, the multi-temporal study highlights the dynamic nature of urban spatial form change, which is conducive to identifying trends in urban landscape and construction patterns in the context of evolving urban sprawl and construction activities. Although the results of this paper show that changes in the landscape pattern of some built forms are negatively correlated with changes in LST, this does not mean that these built environments can be increased indefinitely in the city, but rather that these types of built environments are more conducive to mitigating the influence of the UHIE, relative to those built environments positively correlated with changes in LST.

#### 4.6.3 Application of combining multiple prediction models in urban thermal environment simulation

The choice of model is crucial to the accuracy of LST prediction. Traditional global and regional models have low resolution, which determines they are not suitable for understanding local LST changes (48). In this paper, a combination of a linear regression model based on curve-fitting prediction and a nonlinear machine learning model based on RF prediction was applied to the simulation of LST, and the dynamic changes of the multi-temporal series of LST historical data were also considered. The multi-model integration approach can improve the accuracy of LST prediction by giving full play to the advantages of different models, thus better presenting the characteristics of the future thermal environment. When selecting the best prediction model, the applicability, advantages, and disadvantages of different models should be considered comprehensively, which helps reduce the overfitting or underfitting problems faced by a single model. This integrated approach is expected to provide new insights for urban thermal environment prediction studies.

#### 4.6.4 Urban planning and thermal environment improvement strategies

LCZ mapping plays an irreplaceable role in comprehensively assessing the urban thermal environment and explaining the spatial distribution of LST (49). The LCZ of study area was reclassified into four types based on the changes in LCZ from 2010 to 2020, and the optimal layout of urban spatial form and thermal environment improvement strategies was proposed from the perspective of urban planning based on the results of the paper:

1. Urbanization type (natural to built-up LCZ). Urbanization is the main driver of UHIE. If the intensity of land development in urban growth cannot be avoided, adjusting the landscape patterns of urban spatial forms can help improve the UHIE. Planning of open, mid-rise buildings in new urban areas is encouraged. When necessary, the average height and footprint of open buildings should be reduced to decrease heat-absorbing surfaces. Optimizing the urban building form by appropriately increasing the average building height of dense buildings, promoting verticalization. Additionally, reduce the AI and Cohesion of dense high-rise buildings, and appropriately increase their AREA_MN and LPI on the outskirts of the city, is recommended.
2. Ecotype (built to natural LCZ). Ecotype is an effective way to improve UHIE. The results show the LST of natural-type LCZ is lower than that of built-up LCZ, and all the natural environment changes are negatively correlated with the LST. Cities are encouraged to increase the natural-type urban spatial form as well as their landscape patterns, especially the AREA_MN and LPI of LCZA, and the PD and AREA_MN of LCZG, to increase the ecosystem’s mitigating capacity for LST. In addition, for natural environment types such as LCZD and LCZE, which are represented by cultivated land and hard-paved areas with significant warming trends, increasing vegetation cover or use cooling materials are strongly needed to reduce heat absorption on hard surfaces.
3. Static (unchanged built-up and natural LCZ): The maintenance of the ecological balance and environmental quality of the city and the protection of natural landscapes and ecosystems from degradation and destruction is suggested. Due to the requirements of urban development and planning intensity, high-density building clusters are easy to form in areas with good landscapes, and the relevant authorities should strictly control the planning boundaries of no-build and limited-build zones (50) and set appropriate development intensities and land-use types.
4. Conversion (LCZ with changes within the built): Conversion type mainly refers to the regeneration of stock, and urban regeneration requires careful consideration of building coverage and building height. Aerial height is achieved without affecting building density (51). Existing dense buildings and built environment types with high heat release should be renewed as much as possible, especially LCZ3 and LCZ10. Cooling materials can be used to retrofit existing buildings for energy efficiency, and built environments with hard surfaces should be considered for permeable paving when they are being renewed. In addition, the configuration of open spaces in cities needs to maintain spatial connections with the surrounding natural environment and promote the penetration of cool air into urban areas.

#### 4.6.5 Limitations and future research

This study has achieved certain results in several aspects, but limitations need to be addressed. In terms of remote sensing data processing, the current remote sensing image classification based on the WUDAPT method can characterize the spatial distribution and quantitative features of urban form more accurately, but the recognition accuracy of single grid can still be improved. Future studies may consider combining multisource data (52, 53), machine learning (54), or boosting the number of training sets as well as postfiltering processing of LCZ maps (55) to improve the LCZ classification accuracy. In analyzing the correlation between changes in urban spatial patterns and LST, only representative, typical LCZ types were selected, and the changes of each of the 16 LCZ types were linked to LST, which will provide a more detailed, comprehensive understanding.

## 5 Conclusion

This study aimed to explore the changes in the spatial form of Chengdu and their effect on LST, and predicted the future characteristics of the urban thermal environment. Based on the research findings, targeted policy recommendations were proposed to provide feasible guidance and references for policy makers in relevant fields. We monitored the changes of urban spatial forms in Chengdu from 2010 to 2020 based on a systematic LCZ zoning scheme and analyzed the relationship between LCZ and LST. STC and emerging hotspot analysis were applied to reveal the spatiotemporal patterns of LST across different LCZ, and an OLS model was used to explore the DR between landscape pattern changes and LST, revealing the spatial distribution of urban landscape pattern changes closely associated with rising LST. A multi-model integration approach was applied to simulate the LST in 2025. We concluded as follows: the change of spatial form in Chengdu has gone through three different stages: urban expansion in the early stage, most typically the transformation of LCZA to LCZ9, the coexistence of urban expansion and renewal in the middle stage, where buildings start to develop in the direction of densification and verticalization, and the upgrading of urban functions and ecological transformation in the later stage, which is manifested in the intensive development of the city and the increase of green coverage. The landscape pattern changes of dense buildings are greater than that of open buildings, and the landscape pattern changes of water are the most stable.

The LST of the built-up LCZ is higher than that of the natural-type LCZ. Under similar building heights, the LST of dense buildings is higher than that of open buildings as a whole. In dense buildings, the LST follows the order low-rise > mid-rise > high-rise buildings. In open buildings, the LST follows the order high-rise > mid-rise > low-rise buildings. Oscillating and new hotspots are the two main spatiotemporal hotspots patterns of LST, accounting for 51.99 and 11.44% of the study area, respectively. The effects of landscape pattern changes on LST varies across different built environment types and time periods, while changes in natural environment landscape patterns are always negatively correlated with LST. Changes in AREA_MN and PD that affect the rise in LST are mainly distributed in the city center. The influence of LSI, AI, Cohesion, and PLAND is more widespread, while the distribution range of LPI is the smallest. The average LST in 2025 will increase by 0.15°C from that of 2020. More low and sub-medium temperature zones will transition to medium temperature zones, while the coverage of high and sub-high temperature areas will shift towards the city center.

## Data availability statement

The original contributions presented in the study are included in the article/supplementary material, further inquiries can be directed to the corresponding author.

## Author contributions

LJ: Writing – review & editing, Writing – original draft, Visualization, Software, Resources, Methodology, Data curation, Conceptualization. XX: Writing – review & editing, Supervision, Project administration, Funding acquisition, Formal analysis, Conceptualization. YW: Writing – review & editing, Validation, Resources, Investigation, Formal analysis. XL: Writing – review & editing, Validation, Resources, Investigation. YZ: Writing – review & editing, Validation, Resources, Investigation. QY: Writing – review & editing, Validation, Investigation, Data curation.

## Footnotes

^1^ http://www.usgs.gov/

^2^ https://data.cma.cn/
